# Intrinsic reconstruction of ice-I surfaces

**DOI:** 10.1126/sciadv.abb7986

**Published:** 2020-09-11

**Authors:** N. Kawakami, K. Iwata, A. Shiotari, Y. Sugimoto

**Affiliations:** Department of Advanced Materials Science, The University of Tokyo, 5-1-5 Kashiwanoha, Kashiwa, Chiba 277-8561, Japan.

## Abstract

Understanding the precise atomic structure of ice surfaces is critical for revealing the mechanisms of physical and chemical phenomena at the surfaces, such as ice growth, melting, and chemical reactions. Nevertheless, no conclusive structure has been established. In this study, noncontact atomic force microscopy was used to address the characterization of the atomic structures of ice Ih(0001) and Ic(111) surfaces. The topmost hydrogen atoms are arranged with a short-range (2 × 2) order, independent of the ice thickness and growth substrates used. The electrostatic repulsion between non–hydrogen-bonded water molecules at the surface causes a reduction in the number of the topmost hydrogen atoms together with a distortion of the ideal honeycomb arrangement of water molecules, leading to a short-range–ordered surface reconstruction.

## Teaser

Atomic force microscopy showed that the reconstruction of ice-I surfaces occurs with a short-range (2 × 2) H-atom order.

## INTRODUCTION

The atomic structure of the surface of a crystal solid is difficult to determine, even if the bulk structure is perfectly identifiable. The lack of adjacent atoms or molecules at a truncated surface destabilizes the atomic structure of the ideal (bulk-like) surface, which can cause surface reconstruction (i.e., modification of the atomic position, periodicity, and chemical composition of the surface). Identifying the surface structure at subnanoscale is the first step in understanding the characteristic chemical and physical properties exhibited at the surface. However, the surface structures of ice—a ubiquitous solid—are still not well-understood.

**Fig. 1:**
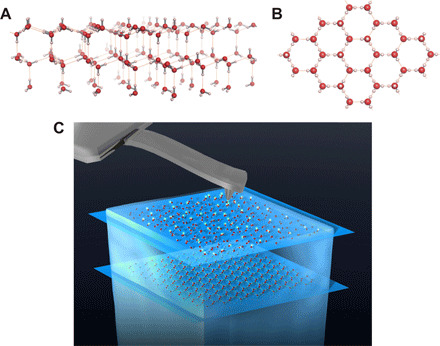
Structure of the ideal surface of crystalline ice Ih. (**A**) Side-view and (**B**) top-view illustrations of the ideal ice-Ih(0001) surface. The arrangement of water molecules in the bulk is restricted by the Bernal-Fowler rules (*2*), in which each an O atom forms two covalent and two hydrogen bonds with four neighboring H atoms tetrahedrally. Therefore, at the ideal surface, the dangling H atoms are distributed randomly with a density of 2.8 atoms/nm^2^. Note that the ideal ice-Ic(111) surface has the same structure despite the difference in the BL stacking manner (*1*). (**C**) Scheme of the ncAFM observation for ice. Red and white spheres represent O and H atoms, respectively.

The presence or absence of the surface reconstruction of ice Ih, which is a representative phase of crystalline ice that appears below the normal pressure (*1*), is still controversial. In the ideal surface of ice Ih(0001), the O atoms of water molecules form a buckling hexagonal lattice, namely, a bilayer (BL). In bulk ice Ih, H atoms have various possible arrangements, even under the “Bernal-Fowler rules” (*2*). At the ideal surface, non–H-bonded H atoms directed toward the vacuum (dangling H atoms) are not perfectly ordered but rather are randomly distributed over the top position of 50% of the topmost O atoms (Fig. 1, A and B). Previous diffraction studies showed (1 × 1) patterns and proposed that the ideal structure was preserved (*3*, *4*), whereas several spectroscopic experiments indicated the existence of surface reconstruction (*5*–*8*). The atomic structure of the ice surface has not been identified despite intensive surface-sensitive experimental measurements (*9*–*11*).

To resolve this serious discrepancy, scanning probe microscopy can provide complementary information to the above-mentioned studies. Although scanning tunneling microscopy (STM) is widely used for the atomic-scale imaging of thin water films (*12*, *13*), it cannot be applied to insulating thick ice. Alternatively, noncontact atomic force microscopy (ncAFM) should be suitable because it can probe insulating materials with atomic resolution (*14*), and it is advantageously sensitive to dangling H atoms at surfaces (*15*–*17*). Although constant-height ncAFM imaging with a carbon monoxide–functionalized tip (*18*) helps visualize individual water molecules in ultrathin water films on metal surfaces (*19*–*21*), thick ice layers fully covered in a substrate disable the tip apex from well-controlled functionalization. A previous ncAFM study with a quartz tuning-fork sensor did not report atomic resolution for thick ice layers grown on Pt(111) (*22*). Therefore, an optimal force sensor is required to visualize the atomic structures of ice surfaces. Using a Si cantilever with an optical interferometer (*23*–*25*), which has greater sensitivity in its interatomic force detectability, is one of the best solutions. Using this force detection method, we conducted ncAFM at 85 K and realized atomic-resolution imaging of ice-I surfaces (Fig. 1C).

## RESULTS

### Visualization of ice-I surfaces

**Fig. 2:**
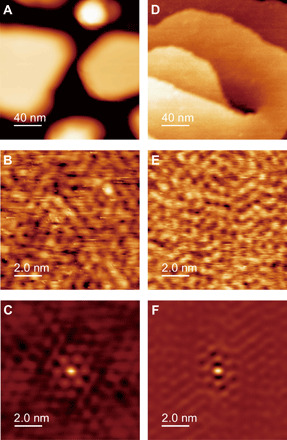
ncAFM images of ice-Ih layers growth on Rh and Pt substrates. (**A**) Overview and (**B**) atomic-resolution ncAFM images of 78 BL ice on Rh(111). The normalized frequency shifts γ were (A) −0.15 and (B) −0.55 fN m^1/2^. (**C**) SC image of (B). (**D**) Overview and (**E**) atomic-resolution ncAFM images of 500 BL ice on Pt(111) [γ = (D) −7.9 and (E) −1.58 fN m^1/2^]. (**F**) SC image of (E).

To grow single-crystalline ice layers, we used two types of typical substrates: single-crystalline Rh(111) and Pt(111) (figs. S1 and S2). On Rh(111), ice layers were grown like hexagonal pillars with a difference in height (Fig. 2A), whereas ice layers homogenously covered the Pt substrate (Fig. 2D) in good agreement with the literature (*26*–*29*). In contrast to the morphological difference, ncAFM images of the terraces for both ice layers show a rather similar appearance (Fig. 2B for Rh and Fig. 2E for Pt). The protrusions in the topographic images reflect an attractive interaction between the tip and sample; thus, they correspond to the positions of the atoms protruded toward the vacuum, i.e., dangling H atoms (*15*–*17*). The H atoms appear to be randomly distributed, just as on the ideal surface (Fig. 1B); however, careful analysis of the AFM images shows the following characteristics and reveals that the surface was reconstructed.

1) Reduction in the number of dangling H atoms. According to the atomic-resolution images shown in Fig. 2 (B and E), the densities of dangling H atoms were calculated to be 1.2 and 1.5 atoms/nm^2^, respectively, and their values are approximately half of the density for the ideal surface (2.8 atoms/nm^2^). The dangling H atoms at these surfaces are more sparsely distributed than those at the ideal surface (Fig. 1B).

2) Short-range (2 × 2) order of dangling H atoms. Although the protrusions in the AFM images seem disordered, the corresponding self-correlation (SC) images exhibit a blunt hexagonal pattern (Fig. 2, C and F). This indicates a short-range order of dangling H atoms. The hexagonal arrangement of the spots suggests that single-crystalline ice was grown in accordance with the substrate atomic lattice (*4*, *30*). The distance of the spots from the center is 0.9 nm for both samples, which is twice that of the O lattice of the ideal ice-Ih(0001) surface (0.446 nm). The (2 × 2) order reasonably explains half the H-atom density relative to the ideal surface. A weak (2 × 2) pattern was previously observed in a diffraction study (*4*), which is likely ascribed to this H-atom order at the surface (see the Supplementary material).

3) Distortion of the O lattice. The protrusions in the images cannot overlap the ideal honeycomb mesh of the O atoms, implying that the ideal lattice of honeycomb O atoms at the surface was distorted. The blunt hexagonal pattern in the SC images was reproduced by a slightly displaced array of spots rather than the perfect (2 × 2)–arranged spots (fig. S7). Note that the (1 × 1) diffraction patterns in the previous diffraction studies (*3*, *4*) probably reflect the distorted O lattice (see the Supplementary material).

**Fig. 3:**
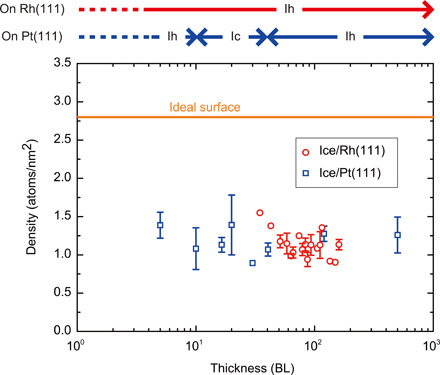
Ice-thickness dependence of the density of dangling H atoms. The orange line indicates the value of the ideal surface. The crystalline phases of ice on the substrates are labeled at the top of the graph.

As shown in Fig. 2, we confirmed that the atomic structure of ice-Ih surfaces is independent of the growth substrates. Furthermore, we observed ice surfaces with various thicknesses on both substrates (Fig. 3) to clarify the effect of the BL stacking manner (i.e., in the comparison between ice Ih and Ic) and H-atom orientation in the bulk (the comparison between para- and ferroelectricity) on the surface reconstruction. In general, ice-Ih exhibits paraelectricity because of the disorder of the H-atom orientation (Fig. 1A). Ice on Rh(111) retains its intrinsic property (*7*, *26*, *27*); however, at the ice-Pt(111) interface, H atoms tend to point toward the substrate, causing ferroelectricity (*9*, *29*). The polarization of the H-atom orientation decays with the ice BL distance from the interface (*9*, *29*). Moreover, the crystalline phase of ice grown on Pt(111) can change, depending on the thickness (*22*, *31*); ice-Ic layers form at medium thicknesses (10 to 50 BLs), whereas ice-Ih layers dominate at other thicknesses (the top panel of Fig. 3; see also fig. S3). Despite the difference in the properties of the bulk, the image appearance is quite similar at any thickness of the ice layer grown on either Rh or Pt substrate, and the corresponding SC image exhibits a (2 × 2) order (figs. S4 to S6). The density of dangling H atoms that was calculated from the topographic ncAFM images (Fig. 3) is independent of the ice thickness and substrate (~1.4 atoms/nm^2^). This result indicates that surface reconstruction is common between ice-Ih(0001) and Ic(111) and robust against changes in the H-atom orientation in the bulk and all interface effects.

### Plausible structural models

**Fig. 4:**
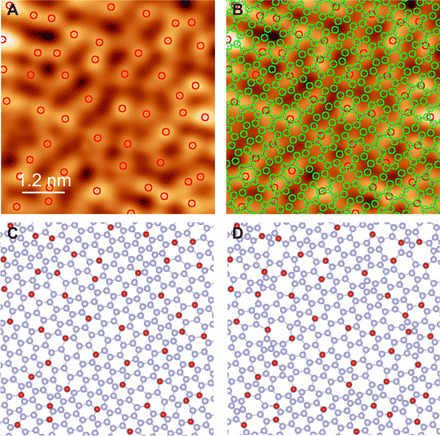
Possible structural models of the ice Ih(0001) surface. (**A**) Atomic-resolution image of 500 BL ice on Pt(111) (γ = −3.57 fN m^1/2^). The signal-to-noise ratio was improved by averaging 20 images of the same area. Red circles represent the positions of the protrusions. (**B**) The same image as in (A) but overlaid by green circles representing O atoms of the ideal lattice. (**C** and **D**) Possible models of surface atomic structures constructed from the image in (A), which have the distorted O lattice without (C) and with (D) heptagonal and pentagonal rings. Blue and red spheres represent O and dangling H atoms, respectively.

The three characteristics mentioned above are not fulfilled by the existing structural models of ice Ih(0001) (*32*–*34*). The information from the ncAFM images is not sufficient to fully determine the molecular geometries and coordinates at the surface because the O lattice is invisible. Nevertheless, we proposed new structural models for future validation via theoretical calculations. On the basis of the positions of dangling H atoms in an ncAFM image of ice Ih (Fig. 4A), we simulated the displacement of O atoms from the ideal honeycomb lattice (Fig. 4B, green) and constructed two possible models (see Materials and Methods): distorted O lattices without and with heptagonal and pentagonal rings (Fig. 4, C and D, respectively). In these models, we assume that several of the topmost O atoms are located beneath the dangling H atoms (Fig. 4, C and D, red), while the other O atoms are separated from the three neighboring O atoms with proper interatomic distances and angles (Fig. 4, C and D, blue). In the former model (Fig. 4C), the hexagonal network is maintained to the greatest degree possible, which enables the layer to be effectively matched with the underlying layer. However, the origin of the lattice distortion cannot be interpreted from this model. Pedersen *et al.* (*35*) theoretically proposed that water molecules at an ice-Ih(0001) surface rotate exothermically to eliminate the dangling H atoms, and this process causes the distorted O lattice with heptagonal and pentagonal rings near the rotated water molecule. The observed surfaces can be interpreted as the result of such a reordering process dominating throughout the surface, causing the number of H atoms to be reduced by half. Therefore, the latter model (Fig. 4D) is more plausible; the hexagonal lattice is mostly maintained, while the rearrangement of the water molecules to reduce dangling H atoms creates a limited number of defects.

## DISCUSSION

We conclude that the reconstruction of the ice-I surfaces originates from the electrostatic repulsion between the dangling H atoms. Several theoretical studies (*32*–*35*) proposed that the electrostatic repulsion between partially charged H atoms plays an important role in the surface structure. The repulsion can be eliminated either by removing of the dangling H atoms (*34*, *35*) or by sparsely distributing the residual dangling H atoms (*33*). Both effects are responsible for the formation of the (2 × 2) H-atom order. The independence of the atomic structure from ice thickness and growth substrate implies that this structure is universal for crystalline-ice surfaces. The reconstruction of the molecular arrangement would play a crucial role in the chemical and physical phenomena occurring at the surfaces, such as melting, ice growth, and chemical reactions (*10*, *36*, *37*).

## MATERIALS AND METHODS

### Materials

We used single-crystalline Pt(111) and Rh(111) substrates (MaTecK) to grow crystalline ice under ultrahigh vacuum (UHV) conditions. The Pt (Rh) substrate was cleaned by several cycles of annealing at 1200 K and Ar^+^ sputtering at 850 (800) K, followed by annealing at 950 (900) K under O_2_ pressure to remove contaminant C atoms from the surface. The growth of crystalline ice was performed by dosing H_2_O gas onto the clean substrate. Ultrapure H_2_O was purified via freeze-and-pump cycles, and the gas was injected into the UHV chamber through a tube nozzle, which is separated from the substrate by 10 cm. For ice on the Pt (Rh) surface, the deposition rate was 3 (2) BL/min, and the substrate temperature was maintained at 145 (141) K. In addition, the samples were postannealed. Although we observed several samples under different postannealing parameters (temperature of 110 to 120 K and duration of 10 min to 10 hours), the results were identical.

### Experimental methods

All the experiments were performed in a UHV chamber under a pressure of 5.0 × 10^−10^ torr at 85 K (UNISOKU-based custom-built ncAFM/STM). The details of the system were described elsewhere (*38*). The STM observation was performed with a PtIr tip (UNISOKU) in constant current mode. The ncAFM measurements were conducted in frequency modulation mode using Si cantilevers (BudgetSensors). Typical values of the stiffness *k*, eigenfrequency *f*_0_, and *Q* factor for the cantilevers were 30 N/m, 3 × 10^5^ Hz, and 1 × 10^5^, respectively. The oscillation of the cantilever was measured by an optical interferometer. We note that the signal-to-noise ratio in our Si cantilever–based ncAFM is larger than that in the quartz tuning fork sensor (qPlus)–based ncAFM by two orders of magnitude (*23*). Oscillation amplitude *A* of the cantilever was kept at 10 nm during the measurement. The ncAFM images were obtained in constant frequency-shift mode. The set point for each image was described as the normalized frequency shift

$$\gamma =\frac{{\mathrm{kA}}^{\frac{3}{2}}}{f_{0}}\Delta f$$ (1)

where Δ*f* denotes the frequency shift. To minimize the long-range electrostatic force, the average contact potential difference was compensated by applying a bias voltage to the sample during the ncAFM measurement.

### Method for obtaining the atomic structural models of the ice-Ih(0001) surface

The structure models shown in Fig. 4 (C and D) were constructed by the following procedure:

1) An ideal honeycomb O-atom lattice was superimposed on the ncAFM image (Fig. 4B, green).

2) The positions of dangling H atoms were determined from the protrusions in the ncAFM image (Fig. 4, A and B, red).

3) We assumed that a dangling H atom was located just above the nearest neighboring O atom (O_NN_) on the surface BL. Then, O_NN_ for each dangling H was uniquely assigned.

4) This process was skipped for the model without heptagonal and pentagonal rings (Fig. 4C) but was considered for the model with heptagonal and pentagonal rings (Fig. 4D). A Stone-Wales defect (fused 5-7-7-5–membered rings) in the ideal honeycomb lattice was assumed to be located near a dangling H atom. This defect was adopted only when the position of O_NN_ became closer to the dangling H atom than in the absence of the defect.

5) The next-nearest neighboring O atoms for each dangling H (O_NNN_, i.e., three O atoms directly bonded to O_NN_ via hydrogen bonding) were placed around an O_NN_ atom with the ideal O─O length and bonding angle.

6) The positions of O_NN_ and O_NNN_ were fixed. The other O atoms were displaced from the original positions so that each O atom was located at the weight center of its three neighboring O atoms. We repeated this process 10 times.

7) The positions of O_NN_ were fixed. The other O atoms (including O_NNN_) were displaced from the original positions so that each O atom was located at the weight center of its three neighboring O atoms. This operation resolved the large distortion of the bonding between O_NNN_ and its neighboring atoms. We repeated this process until the position of all the O atoms become stable.

We varied the lateral positions of the initial honeycomb structure over the ncAFM image (Fig. 4B) and executed the model calculations. As the most reasonable result, we adopted the atomic structures with the smallest dispersion of bonding angles between O atoms (Fig. 4C).

Processes 5 and 6 ensure that oxygen atoms near dangling H atoms (i.e., O_NN_ and O_NNN_) are as undistorted as possible. We imposed these conditions to express that the distortion was increased in regions where dangling H atoms disappeared due to the rotation of the water molecules (*35*).

## Supplementary Material

abb7986_SM.pdf

## SUPPLEMENTARY MATERIALS

Supplementary material for this article is available at http://advances.sciencemag.org/cgi/content/full/6/37/eabb7986/DC1

## References

[1] P. V. Hobbs, *Ice Physics, Oxford Classic Texts in the Physical Sciences* (Oxford Univ. Press, 1974).

[2] Bernal J. D., Fowler R. H., A theory of water and ionic solution, with particular reference to hydrogen and hydroxyl ions. J. Phys. Chem. 1, 515–548 (1933).

[3] Materer N., Starke U., Barbieri A., Van Hove M. A., Somorjai G. A., Kroes G. -J., Minot C., Molecular surface structure of ice(0001): dynamical low-energy electron diffraction, total-energy calculations and molecular dynamics simulations. Surf. Sci. 381, 190–210 (1997).

[4] Glebov A., Graham A. P., Menzel A., Toennies J. P., Senet P., A helium atom scattering study of the structure and phonon dynamics of the ice surface. J. Chem. Phys. 112, 11011–11022 (2000).

[5] Rowland B., Kadagathur N. S., Devlin J. P., Buch V., Feldman T., Wojcik M. J., Infrared spectra of ice surfaces and assignment of surface-localized modes from simulated spectra of cubic ice. J. Chem. Phys. 102, 8328–8341 (1995).

[6] Nordlund D., Ogasawara H., Wernet P., Nyberg M., Odelius M., Pettersson L. G. M., Nilsson A., Surface structure of thin ice films. Chem. Phys. Lett. 395, 161–165 (2004).

[7] Otsuki Y., Sugimoto T., Ishiyama T., Morita A., Watanabe K., Matsumoto Y., Unveiling subsurface hydrogen-bond structure of hexagonal water ice. Phys. Rev. B 96, 115405 (2017).

[8] Sugimoto T., Otsuki Y., Ishiyama T., Morita A., Watanabe K., Matsumoto Y., Topologically disordered mesophase at the topmost surface layer of crystalline ice between 120 and 200 K. Phys. Rev. B 99, 121402 (2019).

[9] Su X., Lianos L., Shen Y. R., Somorjai G. A., Surface-Induced Ferroelectric Ice on Pt(111). Phys. Rev. Lett. 80, 1533–1536 (1998).

[10] Smit W. J., Tang F., Sánchez M. A., Backus E. H. G., Xu L., Hasegawa T., Bonn M., Bakker H. J., Nagata Y., Excess Hydrogen Bond at the Ice-Vapor Interface around 200 K. Phys. Rev. Lett. 119, 133003 (2017). 29341676 10.1103/PhysRevLett.119.133003

[11] Nojima Y., Suzuki Y., Takahashi M., Yamaguchi S., Proton Order toward the Surface of Ice Ih Revealed by Heterodyne-Detected Sum Frequency Generation Spectroscopy. J. Phys. Chem. Lett. 8, 5031–5034 (2017). 28968104 10.1021/acs.jpclett.7b02198

[12] Shimizu T. K., Maier S., Verdaguer A., Velasco-Velez J.-J., Salmeron M., Water at surfaces and interfaces: From molecules to ice and bulk liquid. Prog. Surf. Sci. 93, 87–107 (2018).

[13] Nie S., Bartelt N. C., Thümer K., Evolution of proton order during ice-film growth: An analysis of island shapes. Phys. Rev. B 84, 035420 (2011).

[14] Giessibl F. J., The qPlus sensor, powerful core for the atomic force microscope. Rev. Sci. Instrum. 90, 011101 (2019). 30709191 10.1063/1.5052264

[15] Enevoldsen G. H., Foster A. S., Christensen M. C., Lauritsen J. V., Besenbacher F., Noncontact atomic force microscopy studies of vacancies and hydroxyls of TiO_2_(110): experiments and atomistic simulations. Phys. Rev. B 76, 205415 (2007).

[16] Yurtsever A., Fernández-Torre D., González C., Jelínek P., Pou P., Sugimoto Y., Abe M., Pérez R., Morita S., Understanding image contrast formation in TiO_2_ with force spectroscopy. Phys. Rev. B 85, 125416 (2012).

[17] Stetsovych O., Todorović M., Shimizu T. K., Moreno C., Ryan J. W., León C. P., Sagisaka K., Palomares E., Matolín V., Fujita D., Perez R., Custance O., Atomic species identification at the (101) anatase surface by simultaneous scanning tunnelling and atomic force microscopy. Nat. Commun. 6, 7265 (2015). 26118408 10.1038/ncomms8265 PMC4491188

[18] Gross L., Mohn F., Moll N., Liljeroth P., Meyer G., The chemical structure of a molecule resolved by atomic force microscopy. Science 325, 1110–1114 (2009). 19713523 10.1126/science.1176210

[19] Shiotari A., Sugimoto Y., Ultrahigh-resolution imaging of water networks by atomic force microscopy. Nat. Commun. 8, 14313 (2017). 28155856 10.1038/ncomms14313 PMC5296746

[20] Shiotari A., Sugimoto Y., Kamio H., Characterization of two- and one-dimensional water networks on Ni(111) via atomic force microscopy. Phys. Rev. Mater. 3, 093001 (2019).

[21] Ma R., Cao D., Zhu C., Tian Y., Peng J., Guo J., Chen J., Li X.-Z., Francisco J. S., Zeng X. C., Xu L.-M., Wang E.-G., Jiang Y., Atomic imaging of the edge structure and growth of a two-dimensional hexagonal ice. Nature 577, 60–63 (2020). 31894149 10.1038/s41586-019-1853-4

[22] Thürmer K., Nie S., Formation of hexagonal and cubic ice during low-temperature growth. Proc. Natl. Acad. Sci. U.S.A. 110, 11757–11762 (2013). 23818592 10.1073/pnas.1303001110 PMC3718116

[23] Iwata K., Yamazaki S., Mutombo P., Hapala P., Ondráček M., Jelínek P., Sugimoto Y., Chemical structure imaging of a single molecule by atomic force microscopy at room temperature. Nat. Commun. 6, 7766 (2015). 26178193 10.1038/ncomms8766 PMC4518281

[24] Kaiser U., Schwarz A., Wiesendanger R., Magnetic exchange force microscopy with atomic resolution. Nature 446, 522–525 (2007). 17392782 10.1038/nature05617

[25] Inami E., Sugimoto Y., Accurate extraction of electrostatic force by a voltage-pulse force spectroscopy. Phys. Rev. Lett. 114, 246102 (2015). 26196989 10.1103/PhysRevLett.114.246102

[26] Otsuki Y., Watanabe K., Sugimoto T., Matsumoto Y., Enhanced structural disorder at a nanocrystalline ice surface. Phys. Chem. Chem. Phys. 21, 20442–20453 (2019). 31502600 10.1039/c8cp07269h

[27] Beniya A., Sakaguchi Y., Narushima T., Mukai K., Yamashita Y., Yoshimoto S., Yoshinobu J., The growth process of first water layer and crystalline ice on the Rh(111) surface. J. Chem. Phys. 130, 034706 (2009). 19173536 10.1063/1.3060952

[28] Thürmer K., Bartelt N. C., Growth of multilayer ice films and the formation of cubic ice imaged with STM. Phys. Rev. B 77, 195425 (2008).

[29] Sugimoto T., Aiga N., Otsuki Y., Watanabe K., Matsumoto Y., Emergent high-Tc ferroelectric ordering of strongly correlated and frustrated protons in a heteroepitaxial ice film. Nat. Phys. 12, 1063–1068 (2016).

[30] Braun J., Glebov A., Graham A. P., Menzel A., Toennies J. P., Structure and Phonons of the Ice Surface. Phys. Rev. Lett. 80, 2638–2641 (1998).

[31] Delzeit L., Devlin M. S., Rowland B., Devlin J. P., Buch V., Brad Rowland, J. P. Devlin, V. Buch, Adsorbate-induced partial ordering of the irregular surface and subsurface of crystalline ice. J. Phys. Chem. 100, 10076–10082 (1996).

[32] Pan D., Liu L. -M., Tribello G. A., Slater B., Michaelides A., Wang E., Surface Energy and Surface Proton Order of Ice Ih. Phys. Rev. Lett. 101, 155703 (2008). 18999613 10.1103/PhysRevLett.101.155703

[33] Buch V., Groenzin H., Li I., Shultz M. J., Tosatti E., Proton order in the ice crystal surface. Proc. Natl. Acad. Sci. U.S.A. 105, 5969–5974 (2008). 18408162 10.1073/pnas.0710129105 PMC2329717

[34] Watkins M., Pan D., Wang E. G., Michaelides A., VandeVondele J., Slater B., Large variation of vacancy formation energies in the surface of crystalline ice. Nat. Mater. 10, 794–798 (2011). 21892176 10.1038/nmat3096

[35] Pedersen A., Wikfeldt K. T., Karssemeijer L., Cuppen H., Jónsson H., Molecular reordering processes on ice (0001) surfaces from long timescale simulations. J. Chem. Phys. 141, 234706 (2014). 25527956 10.1063/1.4903812

[36] Ehrenfreund P., Charnley S. B., Organic molecules in the interstellar medium, comets, and meteorites: a voyage from dark clouds to the early earth. Annu. Rev. Astron. Astrophys. 38, 427–483 (2000).

[37] Bartels-Rausch T., Bergeron V., Cartwright J. H. E., Escribano R., Finney J. L., Grothe H., Gutiérrez P. J., Haapala J., Kuhs W. F., Pettersson J. B. C., Price S. D., Sainz-Díaz C. I., Stokes D. J., Strazzulla G., Thomson E. S., Trinks H., Uras-Aytemiz N., Ice structures, patterns, and processes: A view across the icefields. Rev. Mod. Phys. 84, 885–944 (2012).

[38] Suehira N., Tomiyoshi Y., Sugawara Y., Morita S., Low-temperature noncontact atomic-force microscope with quick sample and cantilever exchange mechanism. Rev. Sci. Instrum. 72, 2971–2976 (2001).

[39] Nie S., Feibelman P. J., Bartelt N. C., Thürmer K., Pentagons and heptagons in the first water layer on Pt(111). Phys. Rev. Lett. 105, 026102 (2010). 20867718 10.1103/PhysRevLett.105.026102

