# Supplementary material for: Intrinsic reconstruction of ice-I surfaces
Source: Sci Adv. 2020 Sep 11;6(37):eabb7986. doi: 10.1126/sciadv.abb7986 (PMC7486089; doi:10.1126/sciadv.abb7986)
Supplement: abb7986_SM.pdf [file abb7986_SM.pdf]

Science Advances
AAAS

advances.sciencemag.org/cgi/content/full/6/37/eabb7986/DC1

Supplementary Materials for

Intrinsic reconstruction of ice-I surfaces

N. Kawakami, K. Iwata, A. Shiotari, Y. Sugimoto*

*Corresponding author. Email: ysugimoto@k.u-tokyo.ac.jp

Published 11 September 2020, *Sci. Adv.* **6**, eabb7986 (2020)
DOI: 10.1126/sciadv.abb7986

This PDF file includes:

Supplementary Text
Figs. S1 to S7
References

Supplementary Text

Confirmation of the substrate cleanness and water adsorption

The cleanness of the substrates was confirmed by scanning tunneling microscopy (STM). Figures S1A and S1B show the overview and atomic-resolution STM images, respectively, of a clean Pt(111) surface. The atomic structure of Pt(111) was also confirmed by a noncontact atomic force microscopy (ncAFM) image in constant frequency-shift mode (Fig. S1D). In the STM images, triangular islands (circle in Fig. S1A) and atomic-scale depressions (circle in Fig. S1B) were observed as typical impurities. Even with the existence of these impurities, crystalline ice layers were successfully formed. Figure S1C shows an STM image of H_2O/Pt(111) at 1.4 monolayers (ML). In the image, protrusions aligned with a periodicity of $\left(\sqrt{39}\times\sqrt{39}\right)R16.1^{\circ}$ appeared, which is assigned to a typical water monolayer on the surface (*39*).

Figures S2A and S2B show STM images of a clean Rh(111) surface. The impurities appearing on Pt(111) were not observed, whereas some protrusions and depressions were observed in the atomic-resolution image (Fig. S2B). Figure S2C shows an STM image of 0.8 ML H_2O on the surface. Small islands with a $\left(\sqrt{3}\times\sqrt{3}\right)R30^{\circ}$ pattern were predominantly observed, which agrees with a previous report (*27*). Thus, we confirmed that the impurities do not affect the formation of water monolayers or the further adsorption of water on the monolayers, i.e., the growth of crystalline ice on the surfaces.

Characterization of ice Ic and Ih grown on Pt(111)

The bulk structures of crystalline ice layers grown on Pt(111) were reported to change with ice thickness. The structures can be discriminated by the shape of the screw dislocations at the surfaces (*22, 28*). Figures S3A and S3C show overview ncAFM images of 20 and 500 bilayers (BL) ice, respectively, grown on Pt(111). The screw dislocations are indicated by blue dashed arrows in the images. A screw dislocation on the 20 BL ice smoothly connects a single step between BLs, whereas that on the 500 BL ice connects a double step. The structural difference originates from the bulk crystalline structure; the smooth connection of a single (double) step indicates that a layer has an equivalent (inequivalent) atomic structure to that of the next upper layer (*22*). Therefore, the 20 and 500 BL ice layers are assigned to ice-Ic and Ih, respectively, in accordance with the thickness dependence reported previously (*22*).

Figures S3B and S3D show line profiles along the red arrows represented in Figs. S3A and S3C, respectively. The line profiles indicate that the step height in both samples is approximately 0.36 nm, which matches well with the spacing of the BLs for crystalline ice Ic(111) and Ih(0001). Therefore, it is reasonable to consider that the bulk is well crystallized, and our experimental results reflect the intrinsic nature of crystalline ice-I surfaces.

ncAFM images of ice-I layers with various thicknesses

As described in the main text, the atomic structure of ice-I surfaces is independent of ice thickness. The surface reconstruction is probably driven by the discontinuation of the H-bonding network at the surface. Stacking further water molecules on the

reconstructed surface would repair the molecular orientation and the distortion of the O lattice, producing a bulk BL with an intrinsic order; instead, the additional water molecules form a reconstructed BL, covering the bulk BL.

In the main text, atomic-resolution ncAFM images of ice-Ih(0001) surfaces of 500 BL ice layers grown on Pt(111) are shown. As shown in Fig. 3, we also observed several layers, including ice-Ic(111) surfaces. Figures S4A and S4C show ncAFM images of 20 and 120 BL ice, respectively, grown on Pt(111). The corresponding self-correlation (SC) images (Figs. S4B and D) show a (2 × 2) order, as with the 500 BL ice. The densities of dangling H atoms were calculated as 1.29 ± 0.39 and 1.26 ± 0.21 atoms/nm^2 at 20 and 120 BL, respectively. As described above (see also Fig. S3 and Fig. 3), the surface of the 20 (120) BL ice is assigned to an ice-Ic(111) (ice-Ih(0001)) surface. Therefore, we concluded that the surface structure is independent of the bulk structure (i.e., Ic or Ih).

In the main text, ncAFM images of 78 BL ice grown on Rh(111) are shown. Here, we additionally show ncAFM images of thinner and thicker ice layers on the substrate (Fig. S5); 30 BL (Figs. S5A and S5B) and 115 BL (Figs. S5D and S5E) H_2O were deposited on clean Rh(111). In the former coverage, ice islands grew on the substrate in a pillar-like manner (Fig. S5A), and the height of the islands was distributed in a range between 33 and 40 BL. The other area was probably covered by ultrathin water layers, and therefore, the average thickness was 30 BL. To obtain the ice-thickness dependence of the dangling-H-atom density (Fig. 3 in the main text), surfaces of individual islands were observed, and the thicknesses were determined by the height of each island. By further deposition of water, ice islands on Rh(111) became larger. At 115 BL, the islands eventually merged with each other (Fig. S5D). The growth behavior is in good agreement with a recent report (*26*). Figures S5B and S5E show high-resolution ncAFM images of surfaces of 35 and 115 BL layers, respectively. They have a similar appearance, and the corresponding SC images (Figs. S5C and S5F) show a (2 × 2) pattern, as in the case of the 78 BL ice (see Fig. 2F in the main text).

We confirmed that the (2 × 2) order in SC images is independent of the ice thickness and metal substrates. Figure S6 shows the lattice constant of the ice surfaces obtained by the spot separations in the SC images. The value is ~0.9 nm, which is double the lattice constant of the ideal ice surface.

Simulations of an ncAFM image of the ice-Ih(0001) surface

To quantitatively evaluate the degree of O-lattice distortion, we simulated the (2 × 2) appearance of experimental SC images, using a different model than that shown in Figs. 4C and 4D. In general, a periodic pattern provides an SC image with sharp spots distributed equally by an interval of the lattice periodicity; meanwhile, a less ordered pattern yields an SC image with blunter spots, and the intensity of the spots decays rapidly with increasing distance from the SC-image center. The SC image derived from the experimental ncAFM images of ice surfaces is ascribed to the latter case.

We simulated an ncAFM image of an ice-Ih(0001) surface as follows.

i. As the initial structure, a perfectly ordered (2 × 2) pattern with a periodicity of 0.892 nm was used. In this pattern, dangling H atoms with a density of 1.4 atoms/nm^2 are located at the corners of the (2 × 2) unit cell. (In this model we used the periodic distance of the ice surface, but we can ignore the atomic structure of the O lattice.) The simulated ncAFM appearance of a dangling H atom is described by a round protrusion of a two-dimensional (2D) Gaussian function. The standard

deviation of the function (0.22 nm) was used to simulate the radius of the protrusions in the experimental image (Fig. 4A in the main text). This provides the simulated image shown in Fig. S7A.

ii. We displaced the dangling H atoms in the (2 × 2) lattice in the following manner. The direction and length of the displacement of each dangling H atom were randomly determined, but the distribution followed a 2D-dimensional Gaussian function. The standard deviation *d* of the Gaussian function was a fitting parameter to reproduce the experimental image. Figure S7B shows an example of the results with *d* = 0.14 nm. The displacement vectors are shown as the red arrows in Fig. S7A.

iii. The SC image was obtained by the simulated image after the H-atom displacement, as shown in Fig. S7C.

Spots in the SC image become blunter with *d*. Figure S7D shows the line profiles along the lattice direction for the simulated SC images with various *d* values. The original (2 × 2) pattern provides a clear oscillation in the SC image, whereas the amplitude was smaller with nonzero *d*. The distortion of the lattice can be estimated by comparing the experimental and simulated SC images.

To directly compare it with the simulated images, an experimental ncAFM image was simplified as follows.

i. To clarify the distribution of dangling H atoms, we used a sharp-contrast image of an ice-Ih(0001) surface (Fig. S7E) that is identical to that shown in Fig. 4A in the main text.

ii. Protrusions (blue circles in Fig. S7E) were replaced by "imitating atoms" (round protrusions exhibiting a 2D Gaussian function with a standard deviation of 0.22 nm). Consequently, we obtained a simplified image (Fig. S7F) and the corresponding SC image (Fig. S7G). This process excludes the height variation in the experimental image. The height of the protrusion may result from the variation in the tilt angle of the dangling H atoms or the variation in the vertical height of the topmost O atoms, owing to the lattice distortion; however, in the simple simulations, we omitted the contribution of the height variation because we cannot identify its origin.

To find the best-fit value of *d*, we compared the line profile in the SC images of the simplified experimental image with simulated ones. The similarity between the two profiles was calculated as

$$D = \frac{1}{1 + \sqrt{\frac{1}{N} \sum_{x=x_{\min}}^{x_{\max}} \left(y_{\text{sim}}(x) - y_{\text{exp}}(x) \right)^2}} \quad , \qquad (2)$$

where $y_{\text{sim}}(x)$ and $y_{\text{exp}}(x)$ denote values of the simulated and experimental line profiles, respectively, and $x_{\max(\min)}$ and *N* are the maximum (minimum) value of the horizontal *x*-axis in the range and number of plots, respectively. The degree of similarity *D* varies between 0 and 1, with higher *D* indicating better matching. Because of the randomness for determining the displacement of the dangling H atoms, *D* differs slightly in each trial, even when using the same *d*. Thus, we conducted 20 trials for each *d* and averaged the *D* values. The results are shown in Fig. S7H. The correlation factor shows a clear dependence on *d*, and the best-fit value is 0.14 nm. This value is equal to the averaged

displacement value relative to the positions of the dangling H atoms located on the pristine O lattice (Fig. S7A). The best-fit value results in the images shown in Figs. S7B and S7C.

Comparison of our structure model with the results of previous diffraction experiments

Previous studies of ice surfaces with low energy electron diffraction (LEED) and He atom scattering (HAS) showed (1 × 1) diffraction patterns (*3, 4*). Therefore, the authors of these studies concluded that the ideal structure was preserved. In contrast, spectroscopic experiments using Fourier transform infrared spectroscopy, X-ray adsorption spectroscopy, and sum frequency generation indicate the existence of surface reconstruction (*5–8*). Although the reconstructed structure of the ice-I surfaces seems to be in conflict with the results of the diffraction experiments (*3, 4*), we can reinterpret the results as follows. In the LEED pattern, blunt spots were observed, which were previously explained by the large vibration of the topmost O atoms (*3*). In fact, the blunt spots were probably caused by the distortion of the O lattice of the reconstructed surface.

HAS is a sensitive method for the topmost surface atoms in particular, and the coherent length of the He atoms in the HAS experiment is approximately 20 nm. The (1 × 1) diffraction spots are ascribed to the topmost O and H atoms at the surface (*4*). The distortion of the O lattice may be responsible for the high background; in fact, a previous HAS report stated that the background intensity in the diffraction scans was relatively high (*30*). In addition to the (1 × 1) pattern, a weak (2 × 2) pattern was observed in the HAS experiment (*4*). Some previous studies have concluded that this (2 × 2) pattern corresponds to the H-atom order at the surface. For example, Buch et al. (*33*) proposed a structural model with dangling H atoms distributed sparsely (the dangling-H-atom density was 2.8 atoms/nm^2), which was derived from a (2 × 1) stripe phase. In contrast, we found that the density was reduced by half (1.4 atoms/nm^2), and thus, we alternatively ascribed the (2 × 2) pattern to the short-range (2 × 2) order of the dangling H atoms.

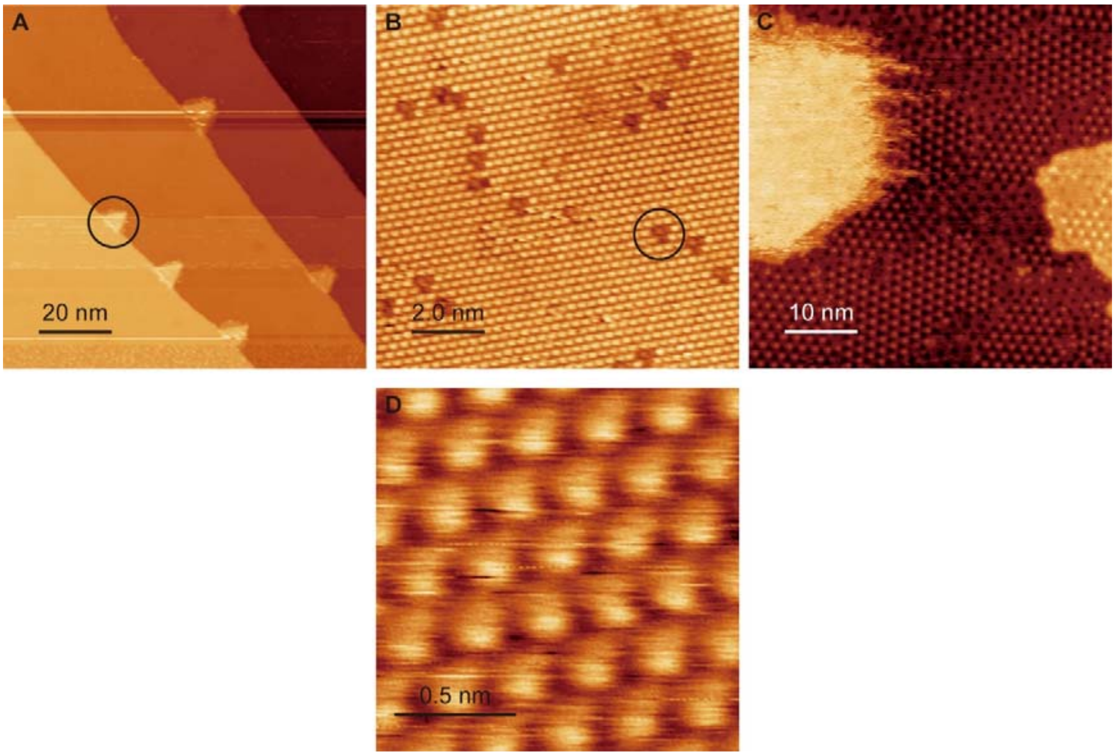

Fig. S1. Clean Pt(111) surface and overlying water monolayers. (**A**) Wide-scale and (**B**) atomic-resolution STM images of a clean Pt(111) substrate. (**C**) STM image of the substrate after depositing 1.4 ML of H_2O. The images were obtained at a sample bias of (A) 0.50, (B) 0.03, and (C) 1.00 V and tunneling current of (A) 0.10, (B) 1.00, and (C) 0.01 nA. (**D**) ncAFM image of a clean Pt(111) substrate ($\gamma = -0.99$ fN m$^{1/2}$).

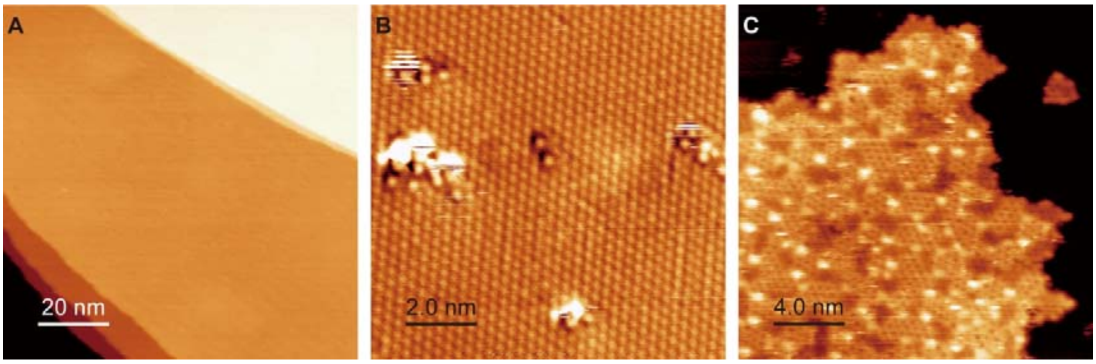

Fig. S2. Clean Rh(111) surface and overlying water monolayers. (**A**) Wide-scale and (**B**) atomic-resolution STM images of a clean Rh(111) substrate. (**C**) STM image of the substrate after depositing 0.8 ML of H_2O. The images were obtained at a sample bias of (A) 1.00, (B) 0.10, and (C) 0.80 V and tunneling current of (A) 0.01, (B) 0.05, and (C) 0.02 nA.

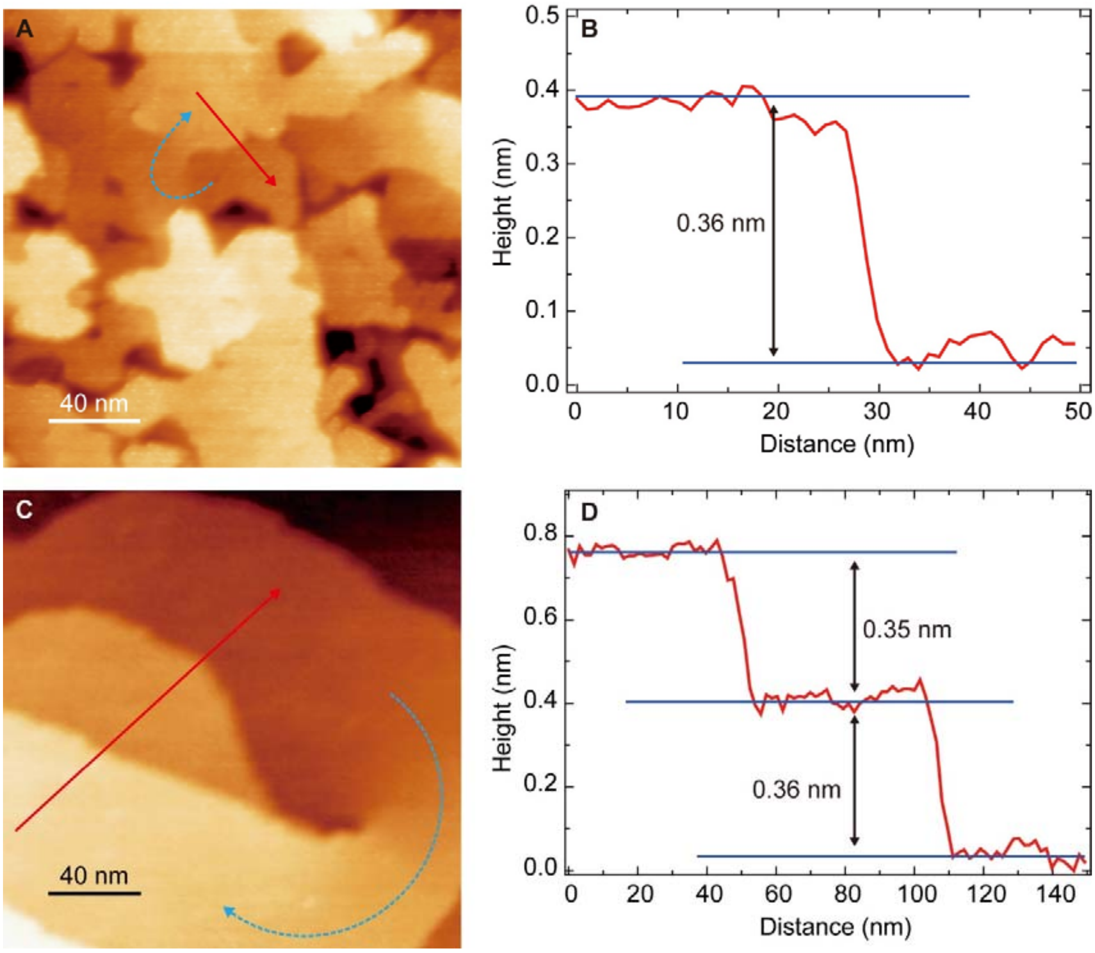

Fig. S3. Characterization of Ice Ic and Ih grown on Pt(111). (**A**) ncAFM image of 20 BL ice grown on Pt(111) ($\gamma = -1.11$ fN m$^{1/2}$). (**B**) Line profile along the red arrow in (A). (**C**) ncAFM image of 120 BL ice grown on Pt(111) ($\gamma = -7.90$ fN m$^{1/2}$). (**D**) Line profile along the red arrow in (C). The blue dashed arrows in (A) and (C) indicate the screw dislocations.

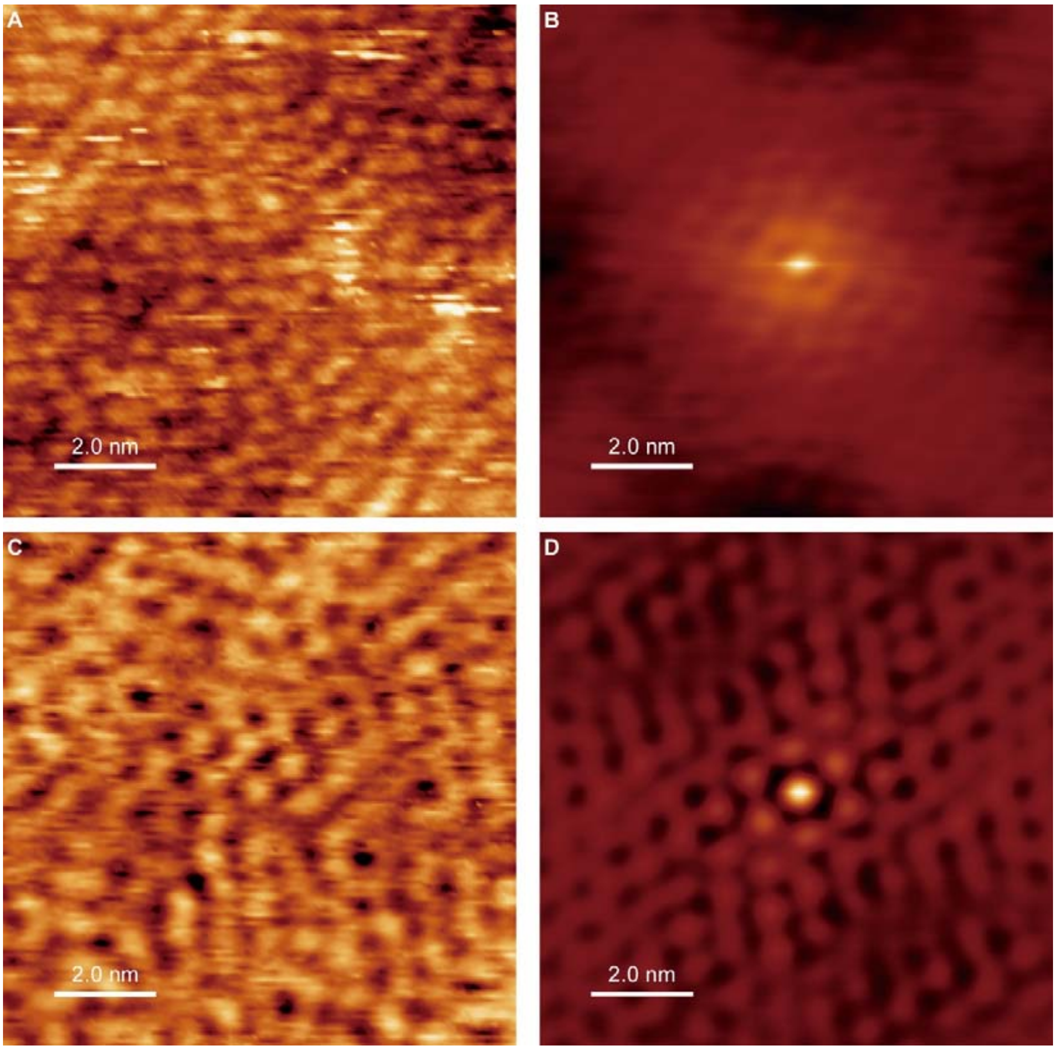

Fig. S4. Ice layers grown on Pt(111). (**A**) Atomic-resolution ncAFM images of 20 BL on Pt(111) ($\gamma = -0.36$ fN m$^{1/2}$). (**B**) SC images of (A). (**C**) Atomic-resolution ncAFM images of 120 BL on Pt(111) ($\gamma = -1.63$ fN m$^{1/2}$). (**D**) SC images of (C).

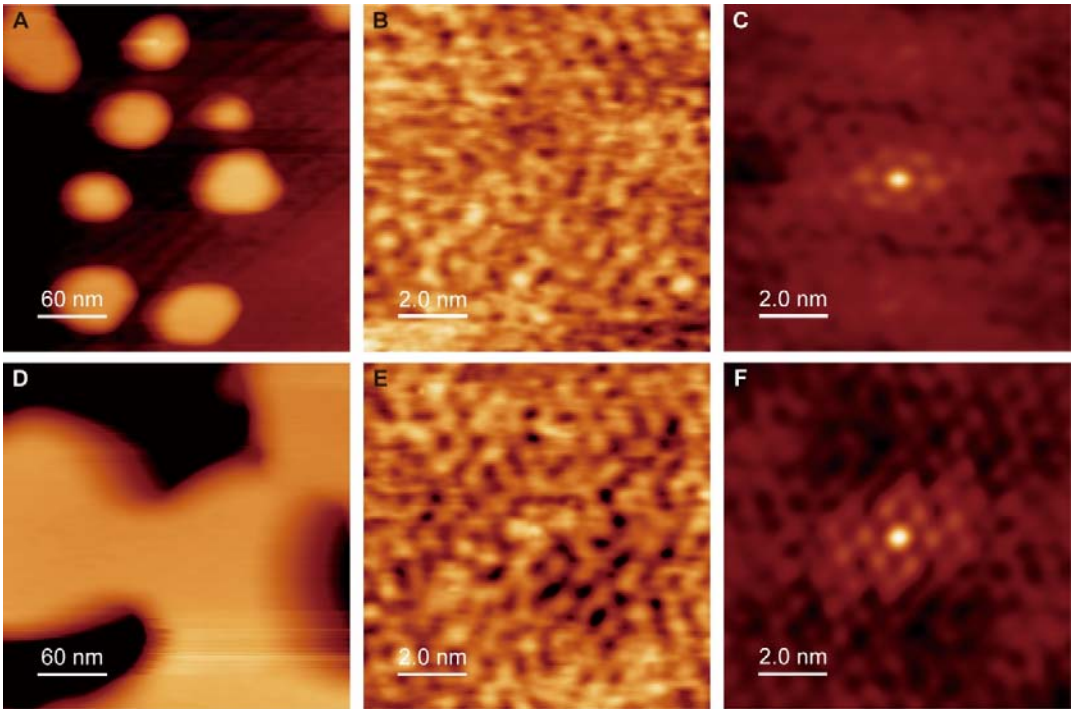

Fig. S5. Ice layers grown on Rh(111). (**A**) Overview ncAFM image of Rh(111) after depositing 30 BL of H_2O ($\gamma = -0.60$ fN $m^{1/2}$). (**B**) Atomic-resolution ncAFM image of a 35 BL ice island ($\gamma = -1.43$ fN $m^{1/2}$). (**C**) SC image of (B). (**D**) Overview ncAFM image of Rh(111) after depositing 115 BL of H_2O ($\gamma = -0.22$ fN $m^{1/2}$). (**E**) Atomic-resolution ncAFM image of a 115 BL ice island ($\gamma = -0.86$ fN $m^{1/2}$). (**F**) SC image of (E).

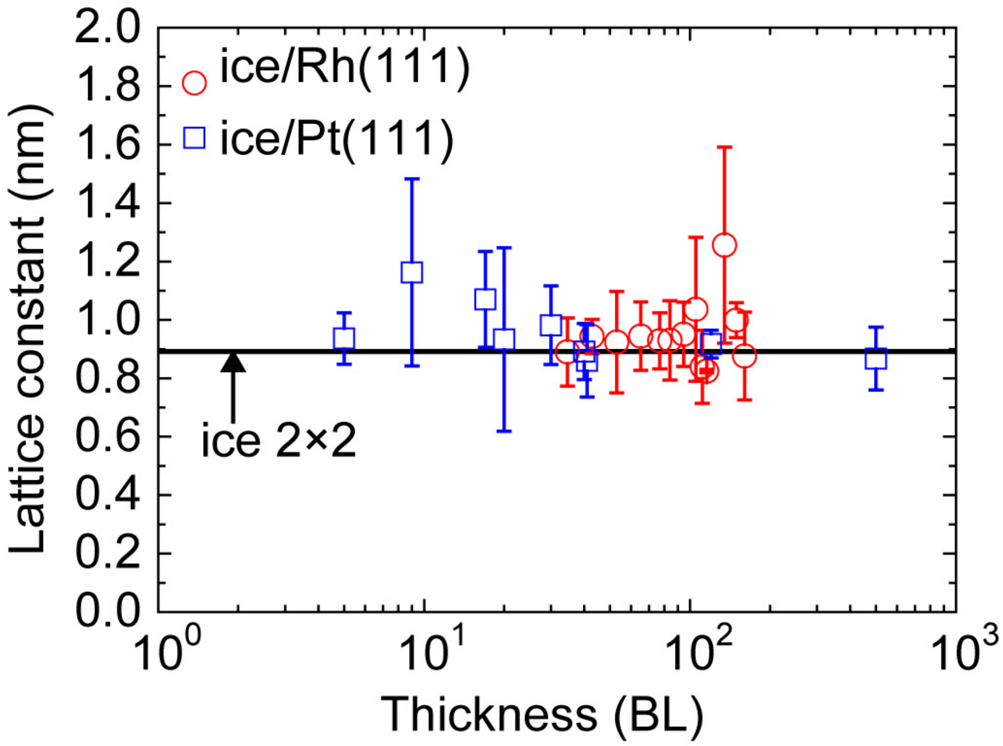

Fig. S6. Ice-thickness dependence of the lattice constant of SC images. Lattice constant of ice surfaces at various ice thicknesses were estimated by the separation between neighboring spots in SC images. The error bar indicates the variation in the images. The black line represents the value of twice the ideal lattice constant of ice Ih(0001).

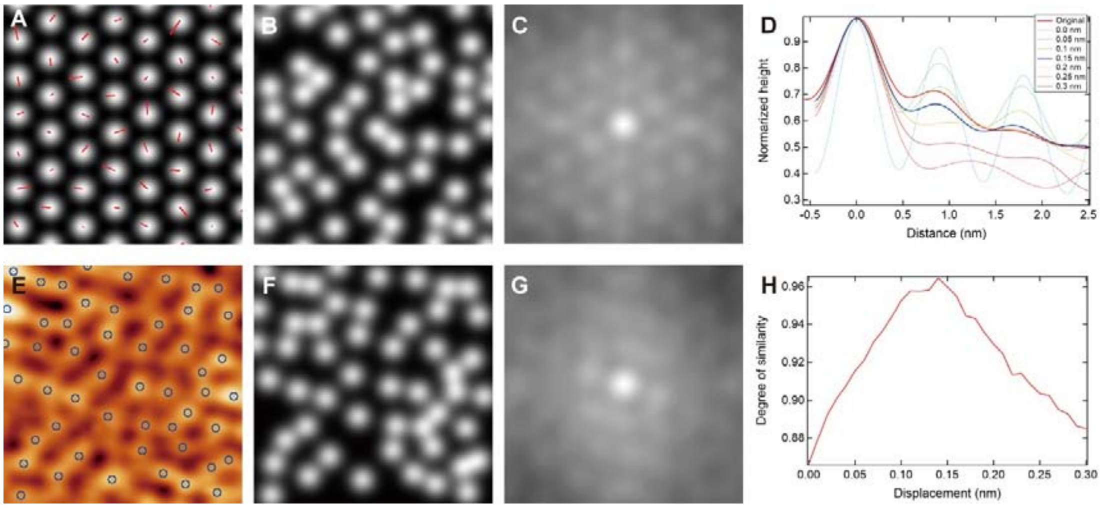

Fig. S7. Simulations to reproduce the short-range order of an ice-Ih surface. (**A**) Simulated image of the initial structure of the ideal (2 × 2) reconstruction, in which the dangling H atoms are depicted as 2D-Gaussian protrusions. The red arrows represent the displacement of the dangling H atoms from the original positions to reproduce the experimental data. (**B**) Simulated image after the displacement. (**C**) SC image of (B). (**D**) Line profiles in SC images of simulated AFM images at various displacement values *d*. The bold red curve represents the experimental line profile. The horizontal axis represents the distance from the SC-image center along the lattice direction. (**E**) Experimental ncAFM image shown in Fig. 4A. (**F**) Simplified image of (E), in which the dangling H atoms are depicted as protrusions described by a 2D Gaussian function. (**G**) SC image of (F). (**H**) Dangling-H-atom-displacement dependence of the similarity between the experimental and simulated images.

REFERENCES AND NOTES

1. P. V. Hobbs, *Ice Physics, Oxford Classic Texts in the Physical Sciences* (Oxford Univ. Press, 1974).

2. J. D. Bernal, R. H. Fowler, A theory of water and ionic solution, with particular reference to hydrogen and hydroxyl ions. *J. Phys. Chem.* **1**, 515–548 (1933).

3. N. Materer, U. Starke, A. Barbieri, M. A. Van Hove, G. A. Somorjai, G. -J. Kroes, C. Minot, Molecular surface structure of ice(0001): dynamical low-energy electron diffraction, total-energy calculations and molecular dynamics simulations. *Surf. Sci.* **381**, 190–210 (1997).

4. A. Glebov, A. P. Graham, A. Menzel, J. P. Toennies, P. Senet, A helium atom scattering study of the structure and phonon dynamics of the ice surface. *J. Chem. Phys.* **112**, 11011–11022 (2000).

5. B. Rowland, N. S. Kadagathur, J. P. Devlin, V. Buch, T. Feldman, M. J. Wojcik, Infrared spectra of ice surfaces and assignment of surface-localized modes from simulated spectra of cubic ice. *J. Chem. Phys.* **102**, 8328–8341 (1995).

6. D. Nordlund, H. Ogasawara, Ph. Wernet, M. Nyberg, M. Odelius, L. G. M. Pettersson, A. Nilsson, Surface structure of thin ice films. *Chem. Phys. Lett.* **395**, 161–165 (2004).

7. Y. Otsuki, T. Sugimoto, T. Ishiyama, A. Morita, K. Watanabe, Y. Matsumoto, Unveiling subsurface hydrogen-bond structure of hexagonal water ice. *Phys. Rev. B* **96**, 115405 (2017).

8. T. Sugimoto, Y. Otsuki, T. Ishiyama, A. Morita, K. Watanabe, Y. Matsumoto, Topologically disordered mesophase at the topmost surface layer of crystalline ice between 120 and 200 K. *Phys. Rev. B* **99**, 121402 (2019).

9. X. Su, L. Lianos, Y. R. Shen, G. A. Somorjai, Surface-Induced Ferroelectric Ice on Pt(111). *Phys. Rev. Lett.* **80**, 1533–1536 (1998)

10. W. J. Smit, F. Tang, M. A. Sánchez, E. H. G. Backus, L. Xu, T. Hasegawa, M. Bonn, H. J. Bakker, Y. Nagata, Excess Hydrogen Bond at the Ice-Vapor Interface around 200 K. *Phys. Rev. Lett.* **119**, 133003 (2017).

11. Y. Nojima, Y. Suzuki, M. Takahashi, S. Yamaguchi, Proton Order toward the Surface of Ice Ih Revealed by Heterodyne-Detected Sum Frequency Generation Spectroscopy. *J. Phys. Chem. Lett.* **8**, 5031–5034 (2017).

12. T. K. Shimizu, S. Maier, A. Verdaguer, J.-J. Velasco-Velez, M. Salmeron, Water at surfaces and interfaces: From molecules to ice and bulk liquid. *Prog. Surf. Sci.* **93**, 87–107 (2018).

13. S. Nie, N. C. Bartelt, K. Thümer, Evolution of proton order during ice-film growth: An analysis of island shapes. *Phys. Rev. B* **84**, 035420 (2011).

14. F. J. Giessibl, The qPlus sensor, powerful core for the atomic force microscope. *Rev. Sci. Instrum.* **90**, 011101 (2019).

15. G. H. Enevoldsen, A. S. Foster, M. C. Christensen, J. V. Lauritsen, F. Besenbacher, Noncontact atomic force microscopy studies of vacancies and hydroxyls of TiO_2(110): experiments and atomistic simulations. *Phys. Rev. B* **76**, 205415 (2007).

16. A. Yurtsever, D. Fernández-Torre, C. González, P. Jelínek, P. Pou, Y. Sugimoto, M. Abe, R. Pérez S. Morita, Understanding image contrast formation in TiO_2 with force spectroscopy. *Phys. Rev. B* **85**, 125416 (2012).

17. O. Stetsovych, M. Todorović, T. K. Shimizu, C. Moreno, J. W. Ryan, C. P. León, K. Sagisaka, E. Palomares, V. Matolín, D. Fujita, R. Perez, O. Custance, Atomic species identification at the (101) anatase surface by simultaneous scanning tunnelling and atomic force microscopy. *Nat. Commun.* **6**, 7265 (2015).

18. L. Gross, F. Mohn, N. Moll, P. Liljeroth, G. Meyer, The chemical structure of a molecule resolved by atomic force microscopy. *Science* **325**, 1110–1114 (2009).

19. A. Shiotari, Y. Sugimoto, Ultrahigh-resolution imaging of water networks by atomic force microscopy. *Nat. Commun.* **8**, 14313 (2017).

20. A. Shiotari, Y. Sugimoto, H. Kamio, Characterization of two- and one-dimensional water networks on Ni(111) via atomic force microscopy. *Phys. Rev. Mater.* **3**, 093001 (2019).

21. R. Ma, D. Cao, C. Zhu, Y. Tian, J. Peng, J. Guo, J. Chen, X.-Z. Li, J. S. Francisco, X. C. Zeng, L.-M. Xu, E.-G. Wang, Y. Jiang, Atomic imaging of the edge structure and growth of a two-dimensional hexagonal ice. *Nature* **577**, 60–63 (2020).

22. K. Thürmer, S. Nie, Formation of hexagonal and cubic ice during low-temperature growth. *Proc. Natl. Acad. Sci. U.S.A.* **110**, 11757–11762 (2013).

23. K. Iwata, S. Yamazaki, P. Mutombo, P. Hapala, M. Ondráček, P. Jelínek, Y. Sugimoto, Chemical structure imaging of a single molecule by atomic force microscopy at room temperature. *Nat. Commun.* **6**, 7766 (2015).

24. U. Kaiser, A. Schwarz, R. Wiesendanger, Magnetic exchange force microscopy with atomic resolution. *Nature* **446**, 522–525 (2007).

25. E. Inami, Y. Sugimoto, Accurate Extraction of Electrostatic Force by a Voltage-Pulse Force Spectroscopy, *Phys. Rev. Lett*. **114**, 246102 (2015).

26. Y. Otsuki, K. Watanabe, T. Sugimoto, Y. Matsumoto, Enhanced structural disorder at a nanocrystalline ice surface. *Phys. Chem. Chem. Phys.* **21**, 20442–20453 (2019).

27. A. Beniya, Y. Sakaguchi, T. Narushima, K. Mukai, Y. Yamashita, S. Yoshimoto, J. Yoshinobu, The growth process of first water layer and crystalline ice on the Rh(111) surface. *J. Chem. Phys.* **130**, 034706 (2009).

28. K. Thürmer, N. C. Bartelt, Growth of multilayer ice films and the formation of cubic ice imaged with STM. *Phys. Rev. B* **77**, 195425 (2008).

29. T. Sugimoto, N. Aiga, Y. Otsuki, K. Watanabe, Y. Matsumoto, Emergent high-Tc ferroelectric ordering of strongly correlated and frustrated protons in a heteroepitaxial ice film. *Nat. Phys.* **12**, 1063–1068 (2016).

30. J. Braun, A. Glebov, A. P. Graham, A. Menzel, J. P. Toennies, Structure and Phonons of the Ice Surface. *Phys. Rev. Lett.* **80**, 2638–2641 (1998).

31. L. Delzeit, M. S. Devlin, B. Rowland, J. P. Devlin, V. Buch, Brad Rowland, J. P. Devlin, V. Buch, Adsorbate-induced partial ordering of the irregular surface and subsurface of crystalline ice. *J. Phys. Chem.* **100**, 10076–10082 (1996).

32. D. Pan, L. -M. Liu, G. A. Tribello, B. Slater, A. Michaelides, E. Wang, Surface Energy and Surface Proton Order of Ice Ih. *Phys. Rev. Lett.* **101**, 155703 (2008).

33. V. Buch, H. Groenzin, I. Li, M. J. Shultz, E. Tosatti, Proton order in the ice crystal surface. *Proc. Natl. Acad. Sci. U.S.A.* **105**, 5969–5974 (2008).

34. M. Watkins, D. Pan, E. G. Wang, A. Michaelides, J. VandeVondele, B. Slater, Large variation of vacancy formation energies in the surface of crystalline ice. *Nat. Mater.* **10**, 794–798 (2011).

35. A. Pedersen, K. T. Wikfeldt, L. Karssemeijer, H. Cuppen, H. Jónsson, Molecular reordering processes on ice (0001) surfaces from long timescale simulations. *J. Chem. Phys.* **141**, 234706 (2014).

36. P. Ehrenfreund, S. B. Charnley, Organic molecules in the interstellar medium, comets, and meteorites: a voyage from dark clouds to the early earth. *Annu. Rev. Astron. Astrophys.* **38**, 427–483 (2000).

37. T. Bartels-Rausch, V. Bergeron, J. H. E. Cartwright, R. Escribano, J. L. Finney, H. Grothe, P. J. Gutiérrez, J. Haapala, W. F. Kuhs, J. B. C. Pettersson, S. D. Price, C. I. Sainz-Díaz, D. J. Stokes, G. Strazzulla, E. S. Thomson, H. Trinks, N. Uras-Aytemiz, Ice structures, patterns, and processes: A view across the icefields. *Rev. Mod. Phys.* **84**, 885–944 (2012).

38. N. Suehira, Y. Tomiyoshi, Y. Sugawara, S. Morita, Low-temperature noncontact atomic-force microscope with quick sample and cantilever exchange mechanism. *Rev. Sci. Instrum.* **72**, 2971–2976 (2001).

39. S. Nie, P. J. Feibelman, N. C. Bartelt, K. Thürmer, Pentagons and heptagons in the first water layer on Pt(111). *Phys. Rev. Lett.* **105**, 026102 (2010).
